# Improving the topical ocular pharmacokinetics of lyophilized cyclosporine A-loaded micelles: formulation, *in vitro* and *in vivo* studies

**DOI:** 10.1080/10717544.2018.1458923

**Published:** 2018-04-10

**Authors:** Yinglan Yu, Daquan Chen, Yanan Li, Wenqian Yang, Jiasheng Tu, Yan Shen

**Affiliations:** a Department of Pharmaceutics, Center for Research Development and Evaluation of Pharmaceutical Excipients and Generic Drugs, School of Pharmacy, China Pharmaceutical University, Nanjing, China;; b School of Pharmacy, Yantai University, Yantai, China

**Keywords:** Cyclosporine A, mPEG-PLA micelles, lyophilized powder, physicochemical characteristics, ocular distribution

## Abstract

Dry eye syndrome (DES) is one of the most common disorders of the eye for which combined treatment includes modification of the ocular environment and pathogenic therapies. Cyclosporine A (CsA), a immunosuppressive agent, has been demonstrated to be effective for the treatment of DES but is limited clinically by its low ocular bioavailability due to poor water solubility. In this paper, methoxy poly (ethylene glycol)-poly (lactide) polymer (mPEG-PLA) micelles were investigated as alternative vehicles for the solubilization and delivery of CsA to the eye. The *in vitro* stability indicated that CsA-loaded micellar lyophilized powder was stable for at least 3 months and the release profile showed a sustained release manner of CsA from micelles physically. *In vivo* ocular distribution studies demonstrated that the micellar formulations exhibited a 4.5-fold increase in retention effect at eyes compared with 0.05% CsA emulsion. In addition, the *in vivo* pharmacokinetics profile showed that the CsA-loaded micelles could enhance the retention time, achieving longer effect toward the DES. These studies proposed an effective micelle formulation as a novel ocular drug delivery system to improve solubility and bioavailability of ophthalmic CsA-controlled delivery.

## 1. Introduction

Dry eye syndrome (DES) is a multifactorial disease of the ocular surface with tear film abnormalities, resulting in tear film stability decrease, eye discomfort, inflammation and lesion of ocular surface (Lemp, 2007). In addition, DES is one of the corneal diseases that cause blindness. It is estimated that the prevalence of DES ranges from about 5% to over 35% in different age groups (Smith et al., 2007), making DES one of the most common ocular conditions (Bjerrum, 1997). Despite its high prevalence, DES is frequently under-recognized (Lin & Yiu, 2014). Owing to its negative influence on patient’s visual function and quality of life, DES causes a big burden in public healthcare. Traditional treatments for DES are focused on increasing lubrication of the ocular surface with artificial tears (Wan et al., 2015). However, these therapies do not address the underlying ocular surface inflammation. Therefore, it is utterly important to identify proper treatments for DES. Currently, a combination of symptomatic therapy, which includes modification of the ocular environment (by increasing humidity, occlusion of lacrimal canaliculi, or simulation of tears), and pathogenic treatments, including the use of antibacterial and anti-inflammatory agents (corticosteroids, antihistamines, tetracyclines and CsA), is currently recommended for DES therapy (Shoji et al., 2005; Kymionis et al., 2008; Moscovici et al., 2012).

Cyclosporine A (CsA) is the anti-inflammatory agent of choice for the treatment of DES, because it can be used long term without adverse effects commonly associated with other anti-inflammatory agents, such as steroids (Utine et al., 2010). Furthermore, unlike corticosteroids, the activity of CsA results from specific and reversible action on T cells, makes it safe for prolonged use (Nussenblatt & Palestine, 1986; Power et al., 1993). However, CsA is a hydrophobic molecule, therefore, is difficult to formulate into conventional topical ocular delivery systems (Ismailos et al., 2011). Currently, commercial topical 0.05% CsA emulsion (Restasis^®^, Allergan) is the only CsA formulation approved by the U.S. Food and Drug Administration (FDA) for the treatment of DES in humans, but it is often accompanied with side effects such as visual disturbance, ocular burning, conjunctival hyperemia, etc (Allergan, 2013). In order to improve the aqueous solubility and permeability of CsA, surfactants or penetration enhancers such as macrogolglycerol ricinoleate and benzalkonium chloride are often used (Furrer et al., 1999). Unfortunately, these additives could typically affect the integrity of the ocular tissues which may lead to high irritation, which limits their further clinical application (Adriaens et al., 2001; Gelderblom et al., 2001; Monti et al., 2002).

Nowadays, significant researches have been performed over recent years to develop safe and effective ocular delivery systems for CsA (Agarwal & Rupenthal, 2016). A variety of novel ophthalmic dosage forms have been explored including in situ gelling systems, hydrogels, nanoparticles, liposomes, and micelles (Agarwal & Rupenthal, 2016). Over the past decade, *in situ* gelling systems have emerged as promising drug delivery systems because of their ease of administration, improved retention, and ability to provide sustained release for a prolonged period of time (Agarwal & Rupenthal, 2013). However, a significant limitation of *in situ* gelling systems might be blurring of vision and burst release because of their increased viscosity, often resulting in higher patient discomfort. In recent years, hydrogels have emerged as a promising alternative for sustained ophthalmic drug delivery because of their ability to prolong corneal residence while being convenient and relatively easy to use (Hu et al., 2011). However, hydrogels are generally not well accepted because of their low transparency and poor oxygen permeability, which blurs the patient’s vision. Nanoparticles have been shown to significantly improve corneal absorption of drugs and accelerate drug penetration, primarily by the transcellular pathway (Mundargi et al., 2008). However, despite their advantages, products based on nanotechnology rarely reach the market as a result of technical issues involved in scale up and manufacturing, as well as the relatively poor scalability and stability of these systems (Vandervoort & Ludwig, 2004). Over the past few decades, liposomes have been used extensively for drug solubilization and targeting because that liposomal formulations tend to accumulate in the cornea to form deposits for sustained delivery over a prolonged period time (Karn et al., 2014). Unfortunately, CsA concentrations in aqueous and vitreous humor after treatment with liposomes prepared by solvent evaporation were low, and could be attributed to the short half-life of the liposomes on the corneal surface, resulting in release of free CsA from the liposomes before they could penetrate all the layers of the cornea (Nikoofal et al., 2013). The use of polymeric micelles is an attractive strategy for improving the corneal and conjunctival penetration of therapeutic drugs, for sustaining drug levels, and reducing systemic side effects (Mondon et al., 2011).

Micelles are self-assembling spherical colloidal systems that are frequently used for the solubilization of hydrophobic molecules. Typically, micelles are formed around a hydrophobic drug in aqueous solution because of the orientation of surfactant molecules to form a hydrophobic core enclosing the drug within a hydrophilic shell (Allen et al., 1999; Kwon, 2003). The methoxy poly (ethylene glycol)-poly (lactide) polymer (mPEG-PLA) is known as the high biocompatible and biodegradable copolymer from FDA and mPEG-PLA micelle has been applied in clinical trials (Panahi et al., 2017). For example, the paclitaxel-loaded mPEG-PLA micelle (Genexol^®^) and paclitaxel loaded mPEG-PDLLA-Phe micelle (Genexol^®^-PM) have approved by FDA. Based on the reported researches, the mPEG-PLA possessed the ability to decrease protein absorption, which has been successfully used for preparing nano-colloid systems as ocular drug delivery systems (Giannavola et al., 2003). It has been reported that micelles self-assembled from mPEG-PLA display enhanced drug penetration, non-irritation to eye and minimal cytotoxicity to cells (Li et al., 2012; Ouahab et al., 2014; Phan et al., 2016).

In spite of many attractive advantages of polymeric micelles as promising drug delivery systems, one drawback is their physical instability. As micelles are in the nanosize range and more dynamic than polymeric nanoparticles, they exhibit greater aggregation tendency due to kinetic motion. Upon storage and transportation, drug leakage may take place due to diffusion of the drug outward the micelles during fluctuation of temperature (Moynihan & Crean, 2009). One approach to overcome such problems is complete elimination of water by lyophilization into a dried powder form. Consequently, their shelf-life can be extended by preventing aggregation of the system and leakage of the loaded drug. Therefore, this study aimed to develop lyophilized cyclosporine A-loaded polymeric micelles using mPEG-PLA as polymers. The physicochemical characteristics of the polymeric micelles were investigated in terms of particle size, polydispersity index (PI), zeta potential, % entrapment efficiency (% EE), and redispersibility. Furthermore, their physical and chemical stabilities after 3 months storage at 4 °C were also evaluated. Finally, the rheological properties, *in vitro* release kinetics, *in vitro* cytotoxicity, cellular uptake, pharmacodynamics and biodistribution of CsA-loaded mPEG-PLA micelles and emulsion were also evaluated.

## 2. Materials and methods

### 2.1. Materials

CsA was purchased from Beijing apuu shilon Biotechnology Co. Ltd. (Beijing, China). mPEG-PLA at different weight ratios (40:60, molecular weighet (MW): 4404.40; 60:40, MW: 3965.50; 80:20, MW: 3155.56) was synthesized by our laboratory previous study (Wang et al., 2017). Acetonitrile and methanol (HPLC grade, 99.9%) were supplied by Tedia Chemical (Fairfield, OH, USA). 3-(4,5-dimethylthiazol-2-yl)-2,5-diphenyltetrazolium bromide (MTT) was provided by Sunshine Biotechnology Co., Ltd. (Nanjing, China). Nile red was purchased from Shanghai Yuyan Biological Technology Co. Ltd. (Shanghai, China). Distilled water made from OPH-II-10 T Ultra pure water system (Sichuan, China). All other reagents were analytical grade from commercial sources and used without further purification.

Human corneal epithelial (HCE-2) cells purchased from the American Type Culture Collection (ATCC^®^ number CRL-11135) were maintained in 75 cm^2^ flasks in Dulbecco’s modified Eagle’s medium (DMEM)/F12 (Gibco, Invitrogen, Carlsbad, Calif., USA) containing 10% fetal calf serum, 100 U/ml penicillin G, and 100 μg/ml streptomycin sulfate in a 37 °C, 5% CO_2_ environment.

New Zealand White rabbits weighing 2-2.5 kg were obtained from Qinglongshan farms (Nanjing, China). All rabbits were healthy and free of clinically observable ocular surface disease. All animal experiments complied with the requirements of the National Institute of Health Guide for Care and procedures were approved by the China Pharmaceutical University Animal Experiment Center.

### 2.2. Preparation and characterization of CsA loaded micelles

#### 2.2.1. Preparation of CsA loaded micelle ophthalmic preparations

In order to form of CsA micelles, the CsA and mPEG-PLA with different weight ratios (40:60, molecular weighet (MW): 4404.40; 60:40, MW: 3965.50; 80:20, MW: 3155.56) were dissolved in 1 ml ethanol solution. It was then vacuum rotary evaporated at 50 °C untill the organic solvent was completely volatile. Finally, physiological saline was added to dissolve the formed transparent colloid and the solution was filtered with 0.22 μm cellulose nitrate membrane to remove the nonencapsulated CsA aggregates.

#### 2.2.2. Formulation and optimization of lyophilized CsA-loaded polymeric micelles

To formulate the dried powder form of CsA-loaded polymeric micelles, the mPEG-PLA with suitable weight ratio was chosen to formulate lyophilized CsA-loaded polymeric micelles. In order to obtain a better appearance, some excipients were added into CsA loaded micelles to provide a lyophilized skeleton. The following six excipients as stabilizers were used in this experiment: glucose, sucrose, L-glutamic acid, sorbitol, mannitol and mPEG2000. 1 ml of CsA loaded micelles and 50 mg stabilizer (5%, w/v) were pipetted into a 5-ml vial and frozen at −75 °C overnight. Subsequently, a vial containing polymeric micelles was submitted to lyophilizer (LGJ-10FD, Beijing Songyuanhuaxing Technology Develop Co., Ltd, China) for at least 24 h. The temperature of the condenser was −40 °C and the pressure was 0.120 mbar. The lyophilized CsA-loaded polymeric micelles were finally collected and kept in the refrigerator until used.

#### 2.2.3. Preparation of CsA oil-in-water (o/w) emulsions

It was a 0.05% (w/v) CsA emulsion of castor oil in water. The lipid phase was prepared by mixing castor oil and Tween 80. CsA dissolved in ethanol was slowly added into the lipid phase. The water phase was prepared by dissolving sodium chloride in water. Sodium chloride (0.9%, w/v) was used to make the emulsion isotonic. After removing ethanol from the lipid phase by N_2_, the water phase and lipid phase were mixed at 10,000 rpm for 5 min using a shaker (MS-100, Hangzhou Allsheng Instruments Co., Ltd., China).

#### 2.2.4. Size distribution and zeta potential

Mean particle size and size distribution of the micelles were measured by an ZetaPlus laser particle size analyzer (Brookhaven Instruments Corporation, USA). The micelles were diluted with physiological saline before the measurement. The zeta potential of the micelles was detected in the same instrument. All measurements were performed in triplicate following dilution of the micelles at 25 °C.

#### 2.2.5. Transmission electron microscopy (TEM)

A drop of CsA micelle solution with the concentration of 0.5 mg/ml was attached to the copper network and was mixed with a drop of 2% (w/v) phosphotungstic acid. The morphology of CsA micelles was examined by transmission electron microscope (JEM-1400, Jeol, Japan).

### 2.3. Percentages of entrapment efficiency

To determine the percentages of entrapment efficiency of the CsA in the micelles, the calculated weight of micelles powder was re-dissolved into 1 ml physiological saline. The micellar solution was diluted with methanol to disrupt the self-assembled structures and the amount of CsA was then determined using High Performance Liquid Chromatography (HPLC, Thermo Fisher, USA). The EE% was calculated by the following formulae:

(1) $$EE\%=\frac{Weight of CsA in micelles}{Weight of the total CsA}\times 100$$

### 2.4. Quantitative analysis of CsA by HPLC

The concentration of CsA was measured by HPLC. HPLC analysis was performed on a reverse C18 column (4.6 × 250 mm, 5 μm, ZORBAX Eclipse Plus C18, Agilent, USA). The mobile phase was a mixture of acetonitrile: water: phosphoric acid (74:26:0.025, v/v/v), filtered through 0.22 μm membrane filter and eluted at a flow rate of 1 ml/min. The UV detector set at 210 nm and the CsA concentration was determined using 20 μl of injection volume at 70 °C.

### 2.5. Redispersibility study of lyophilized CsA – loaded polymeric micelles

The redispersibility of lyophilized polymeric micelles was evaluated according to the previously published method (Suksiriworapong et al., 2014). Physiological saline (1 ml) was slowly added in a lyophilized vial and the lyophilized sample was rehydrated for 1 min. Subsequently, the particle size of the sample was characterized. The results were expressed as the particle size ratio (S_2_/S_1_) according to Equation (2).

(2) $$S_{2}/S_{1}=\frac{Particle size after reconstruction}{Particle size before lyophilization}\times 100$$

### 2.6. Physical stability study of lyophilized CsA-loaded polymeric micelles

The physical stability of lyophilized polymeric micelles was evaluated after 3 months storage at 4 °C. The height of lyophilized cake was compared with that at an initial time. The redispersibility of lyophilized polymeric micelles was conducted according to the aforementioned method. The height of lyophilized cake, particle size, polydispersity index, and zeta potential were recorded after 3 months compared with those at the initial time. The changes in height of lyophilized cake, particle size and polydispersity index of polymeric micelles were expressed as the height (H_3_/H_0_), particle size (S_3_/S_0_), and PI (PI_3_/PI_0_) ratios, respectively. The subscript numbers 0 and 3 denote 0 and 3 months after storage, respectively.

### 2.7. Chemical stability study of lyophilized CsA-loaded polymeric micelles

Based on the physical stability results, the formulations stabilized with 5% (w/v) mPEG2000 were chosen to evaluate their chemical stability in comparison with those without mPEG2000. The chemical stability of the selected lyophilized polymeric micelles was evaluated after 3 months storage at 4 °C. The lyophilized powder was dissolved in 1 ml of physiological saline. The solution was diluted with methanol to disrupt the self-assembled structures and analyzed by HPLC method. The remaining amount of CsA was expressed as % CsA remaining relative to an amount of drug at an initial time.

### 2.8. Rheological studies

The viscosity of the prepared formulations was determined at different shear rate at 37 ± 1 °C using a DHR-2 viscometer (TA Instruments, USA). A typical run involved changing the shear rate from 0.1 to 10 1/s. Evaluations were conducted in triplicate.

### 2.9. *In vitro* release kinetics study

One milliliter of CsA-loaded mPEG-PLA micelles was firstly put in a dialysis bag with 3.5 kDa molecular weight cutoff. After that, the resulting system was incubated in 30 ml sodium lauryl sulfate (SLS) solution (0.25%, w/v) while stirring at 37 °C. Five milliliter of sample was withdrawn at predetermined time intervals and immediately replaced with an equal volume of fresh release medium. The amount of CsA in the sample was analyzed by HPLC as described in Section 2.4. The release study of 0.05% CsA emulsion was also performed for comparison. To evaluate the release mechanism of CsA from mPEG-PLA micelles, the drug release profile was fitted by zero-order, first-order, Higuchi’s, Hixson–Crowell’s, Weibull’s, and Peppas-Sahlin’s models (Siepmann & Peppas, 2001; Dash et al., 2010; Seda Tığlı Aydın & Pulat, 2012). The linear regression analysis was applied for the calculation of *R*. To compare among all models, the adjusted coefficient of determination (*R*_adjusted_) was applied for the determination of the best fit model for the release profiles to describes the best drug release mechanism.

### 2.10. *In vitro* cytotoxicity assay

Cell viability assay was performed to determine the toxicity of CsA, CsA micelles, mPEG-PLA blank micelles and 0.05% CsA emulsion on human corneal epithelial (HCE-2) cells. Briefly, 8000 cells per well were plated into 96-well plates and incubated for 24 h in a humidified atmosphere 5% CO_2_ at 37 °C. The blank micelles, CsA micelles and 0.05% CsA emulsion was diluted to the CsA concentration corresponding to 0.01, 0.1, 1, 5, 10, 20, 50, 100 μg/ml with DMEM medium; the free CsA was dissolved in DMSO at a proper concentration, diluted with DMEM medium to the same concentration and added into the wells. After 24 h and 48 h incubation, the medium was removed and 200 μl of 0.5 mg/ml tetramethylazoles (MTT) was added to each well. The incubation was continued for another 4 h. Then the MTT derivative was dissolved with DMSO and the optical density of the solution was determined by a microplate reader (EL × 800, BioTek, USA) at 570 nm. The cell viability was calculated according to the following formula 3:

(3) $$Cell visability=\frac{I_{\mathrm{sample}}-I_{\mathrm{black}}}{I_{\mathrm{control}}-I_{\mathrm{blank}}}\times 100$$

where *I*_sample_ and *I*_control_ are the mean absorbance value of the tested group and control group, separately. *I*_blank_ is the absorbance value of the medium.

### 2.11. Cellular uptake study

To investigate the effect of mPEG-PLA micelles on the cellular uptake of nile red, nile red labeled micelles and nile red solution with concentration of 1 μg/ml nile red were prepared. To prepare the test samples, nile red and mPEG-PLA were dissolved in 1 ml ethanol to form an organic solution. This solution was dried by vacuum rotary evaporation to obtain transparent colloid. The transparent colloid was then dispersed in 10 ml physiological saline. Finally, the solution was filtered with 0.22 μm cellulose nitrate membrane to get mPEG-PLA micelles solution containing nile red. Nile red solution with the same concentration was also prepared for comparison. The test samples were diluted in PBS to a final nile red concentration of 1 μg/ml. The exact concentrations of nile red in all samples were determined with C18 column (4.6 × 250 mm, 5 μm, ZORBAX Eclipse Plus C18, Agilent, USA), mobile phase, acetonitrile; flow rate, 1 ml/min; excitation and emission wavelength, 550 nm and 600 nm.

Fluorescence microscope was used to assess intracellular uptake efficiency of nile red. HCE-2 cells were seeded directly into 6-well plates at a density of 25 × 10^3^ cells per well. After culturing for 24 h, the culture medium was discarded and the cells were washed with PBS three times. Then, the cells were incubated with test solutions at 37 °C for 4 h. The experiment was stopped by washing the cells three times with PBS to remove non-intracellular drug. The HCE-2 cells were photographed with fluorescence inverted microscope (Olympus IX53IX53, Japan).

### 2.12. Identification of cellular uptake mechanisms

Endocytosis inhibition assay was conducted to verify that how mPEG-PLA micelles were delivered into cells. To study the effect of different inhibitors on the cellular uptake of polymeric micelles, the cells were pre-incubated with different inhibitors for 1 h at 37 °C. Amiloride (133 μg/ml), chlorpromazine (20 μg/ml), and sodium azide (1 mg/ml) were added, respectively. Then the inhibitor containing culture media was discarded and nile red loaded micelles was used for another 2 h incubation. Then the cells were washed with PBS three times to eliminate excess micelles which were not entrapped by the cells. The cells were diluted in 300 μl acetonitrile, vortexed for 5 min and centrifuged for 30 min at 12000 rpm. The uptake was calculated by measuring the content of nile red taken up.

### 2.13. Ocular distribution study

New Zealand white rabbits were divided into 5 groups of 3 rabbits each, and 60 μl of CsA micelles preparation (0.5 mg/ml) and CsA emulsion (0.5 mg/ml) were dropped into the conjunctival sac of each eye, micelles in the right and emulsion in the left. At 1 h, 2 h, 4 h, 8 h and 24 h after drug administration, each rabbit was injected with 20% urethane (1.0 g/kg) through the ear limbus vein, followed by 0.1% 0.1 ml tetracaine instillation into the eye. The surface of the eye was briefly washed with 10 ml 0.9% sodium chloride solution, and the excessive water was blotted with filter paper. An aliquot of 100 μl of the aqueous humor was aspirated from the anterior chamber by paracentesis using a 30-gauge needle attached to a 1 ml syringe. The aqueous humor samples were stored at −20 °C for analysis.

To investigate the distribution in ocular tissues of CsA, rabbits were humanely killed, and their eyes were enucleated at 1 h, 2 h, 4 h, 8 h and 24 h after dosing. The eyes were carefully rinsed with normal saline and dried with filter paper to remove remaining drug. Corneal epithelium was carefully removed using a scalpel. The corneas were excised at the limbus with scissors. All tissues were thoroughly homogenized using a glass homogenizer and then transferred to preweighed tubes. All tubes were weighed again before samples were stored at −80 °C before analysis. For analysis, each sample was mixed with 5 ml diethyl ether. After centrifuging, 20 μl of the supernatant liquid was obtained for HPLC analysis. The concentration of CsA in the supernatant was determined by HPLC and the amount of CsA remaining in per g of the tissues was taken as the concentration of CsA in each time point.

### 2.14. Statistical analysis

The data were analyzed using SPSS software, version 19. Statistical comparison between two individual groups was determined by one-way ANOVA with Bonferroni test. All the experiments in the study were performed at least three times and the data were reported in term of mean and standard deviation (SD).

## 3. Results and discussion

### 3.1. Characterization of CsA micelles

Supplementary Table S1 showed the particle size, zeta potentials and encapsulation efficiency of the micelles prepared with different block ratio of mPEG to PLA. The particle size and EE% of CsA micelles were greatly dependent on the block ratio of mPEG to PLA. With the mPEG/PLA ratio increasing from 80: 20 to 40: 60, the diameter increased from 28.8 nm to 42.2 nm, and the EE% improved from 62.26% to 98.03%, respectively. This phenomenon could be explained as that CsA was insoluble in the aqueous solution. When dispersing the thin film of CsA and mPEG-PLA to an aqueous solution, the CsA and PLA segment served as a hydrophobic core structure while the PEG segment as a hydrophilic shell structure to form a micelle spontaneously. The enlargement of the hydrophobic core and the increasement of the solubilization capacity which result in increasing of particle size and EE% were due to the increasement of the block ratio of PLA. The mPEG-PLA co-polymer with weight ratio of 40: 60 had the appropriate particle size and excellent drug loading capacity, so it was used for further characterization.

**Figure 1.**
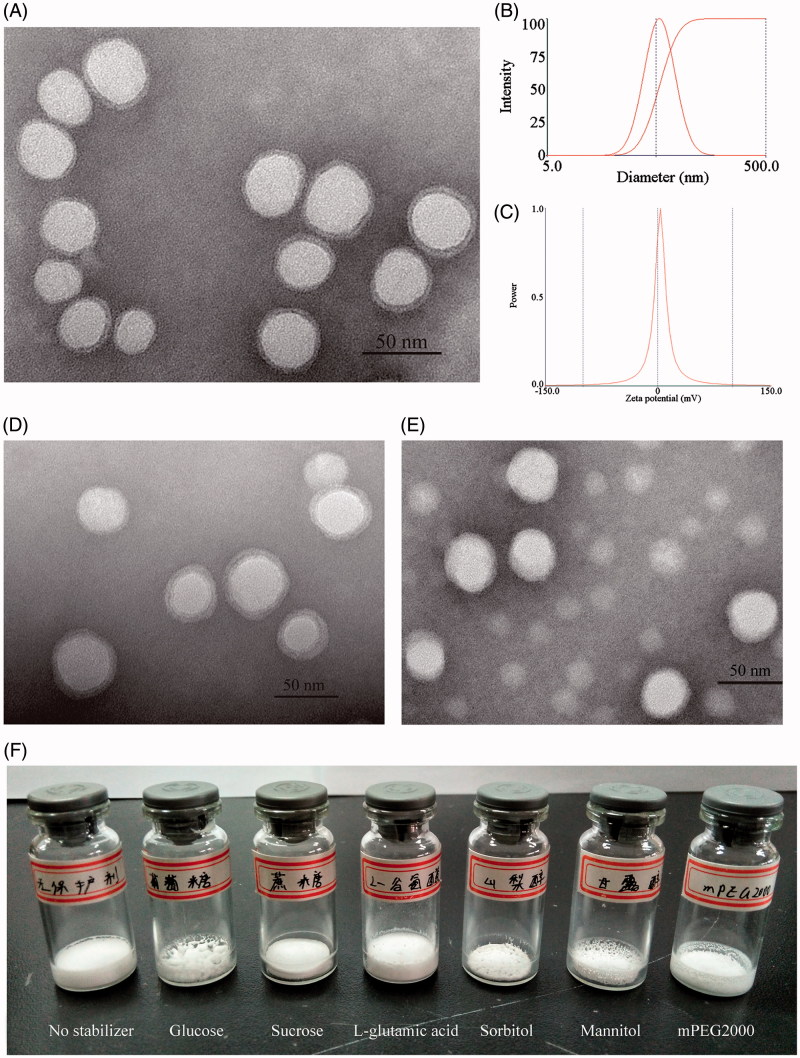
Transmission scanning electron micrographs of CsA loaded mPEG-PLA micelles before lyophilization (A), after reconstitution (D) and after 3 months storage at 4 °C (E); (B) size distribution of CsA-loaded mPEG-PLA micelles; (C) zeta potential of CsA-loaded mPEG-PLA micelles; (F) lyophilized product appearance of six excipients.

The morphology of the obtained micelles was monitored by TEM. As shown in Figure 1(A), we could observe uniform particles with sizes of about 40 nm in the CsA loaded mPEG-PLA micelles and the particles were monodispersed with no aggregations. Most of the micelles were spherical or almost spherical in shape with smooth surface. The image of TEM confirmed that the micelles were spherical core - shell structures. The hydrophobic segments forming polymer micelles could not be stained by phosphotungstic acid. As a result, these parts showed up as bright white cores under the electron microscope. The hydrophilic PEG shells absorbed some of the phosphotungstic acid in the aqueous phase, which displayed as gray halos. The core – shell structures of the micelles could control the drug release and enhance the drug stability (Huang et al., 2014). Furthermore, the zeta potential of CsA-loaded mPEG-PLA micelles was about 1.85 mV (Figure 1(C)).

The desired appearance of lyophilized powder should be an intact and porous cake struture. Furthermore, the color should be uniform. In this experiment, lucose, sucrose, L-glutamic acid, sorbitol, mannitol and mPEG2000 was chosen as a stabilizer for lyophilization of CsA micelles. Then, lyophilized product appearances and the solution appearance after reconstitution were compared, and the results were shown in Supplementary Table S2 and Figure 1(F). By comprehensive comparison, mPEG2000 as the stabilizer was the best because the structure by mPEG2000 as stabilizer was intact and porous, as well as the solution after reconstitution was clear, transparent and stable. After reconstitution, the morphology of the CsA loaded micelles with 5% (w/v) mPEG2000 as a stabilizer was graphically exemplified in Figure 1(D). TEM micrographs indicated that the shape of CsA loaded mPEG-PLA micelles was not changed, still spherical, and the particles were monodispersed with no aggregations. mPEG2000 consists of polyethylene glycol chain structural units and has the similar structure with the micellar shell. The addition of mPEG2000 could make the gap of micelles and micelles filled with polyethylene glycol chain units. In the process of lyophilization, mPEG2000 mainly played the role of steric stabilizers to protect the micelles (Heald et al., 2001).

### 3.2. Physicochemical characterizations

We inspected the crystallinity properties of micelles as well as the possible interaction between drugs and polymers in micelles by FTIR, XRD, and DSC tests. Supplementary Figure S1A shows the FTIR spectra of mPEG2000, the mPEG-PLA co-polymer, CsA powder, a physical mixture of CsA, mPEG-PLA and mPEG2000 as well as lyophilized CsA loaded mPEG-PLA micelles. The spectra of CsA had characteristic peaks of SO^3−^ (1041 cm^−1^) and C = O (1740 cm^−1^) (Konyushenko et al., 2006). The characteristic IR bands of the mPEG-PLA co-polymer consisted of the ones at 1093 and 1134 cm^−1^ belonging to C-O-C of the mPEG and PLA in copolymer, respectively. And absorbance bands at 1761 cm^−1^ were related to the C = O stretching of PLA (Panahi et al., 2017). Comparing the spectrum of freeze-dried CsA loaded mPEG-PLA micelles with that of the physical mixture of CsA, mPEG-PLA and mPEG2000, no new absorption peaks appeared, indicating the absence of any chemical reactions during the sample preparation procedures.

The crystallographic assay was performed by XRD and the results were presented in Supplementary Figure S1B. The XRD for the intact CsA powder showed the specific peaks at 2θ: 6.79°, 7.74°, 8.56°, 9.14°, 9.36°, 10.73°, 12.52°, 14.60°, 15.05°, 15.79°, 16.79°, 19.42° and 20.87°. For the mixture of mPEG-PLA co-polymer and mPEG2000, three characteristic peaks at 2θ: 19.29°, 23.48° and 31.80° could be seen. Both XRD patterns of CsA powder and the mixture of mPEG-PLA and mPEG2000 were in accordance with the previous report (Zheng et al., 2010; Navale et al., 2014). In the XRD pattern of the physical mixture of CsA, mPEG-PLA and mPEG2000, all the characteristic peaks of CsA powder and the mixture of mPEG-PLA and mPEG2000 were observed with decreased intensities, indicating a crystalline state of CsA in the mixture sample. However, the characteristic peaks of CsA were disappeared and only the peaks of mPEG-PLA and mPEG2000 were observed in CsA loaded mPEG-PLA micelles, suggesting that CsA was no longer present as a crystalline state but an amorphous state in the formulation. Similar obervations of amorphous CsA in nanoparticles were documented previously in several studies (Jain et al., 2011). Amorphous pharmaceuticals were markedly soluble than their crystalline counterparts, and for partially amorphous materials the apparent solubility enhancement was likely to influence *in vitro* and *in vivo* dissolution behavior (Hancock & Parks, 2000).

The thermal properties of mPEG2000, mPEG-PLA, CsA powder, the physical mixture of CsA, mPEG-PLA, mPEG2000 as well as lyophilized CsA loaded mPEG-PLA micelles were investigated by DSC measurements. As depicted in Supplementary Figure S1C, the mPEG-PLA co-polymer showed the two expected endothermic peaks at 42.8 °C and 265.6 °C. For the mPEG2000, a sharp endothermic melting peak around 56.8 °C followed by decomposition was observed. For the CsA powder, no obvious endothermic peak or exothermic peak was observed. The characteristic endothermic peaks of the mPEG-PLA co-polymer and mPEG2000 were clearly observed in the DSC curve of the physical mixture of CsA, mPEG-PLA and mPEG2000. In the DSC curve of lyophilized CsA loaded mPEG-PLA micelles, the endothermic peaks of mPEG-PLA were disappeared. All observations suggested the decrease of crystallinity of mPEG-PLA co-polymer and the possible formation of micelles.

### 3.3. Redispersibility study of lyophilized CsA – loaded polymeric micelles

**Figure 2.**
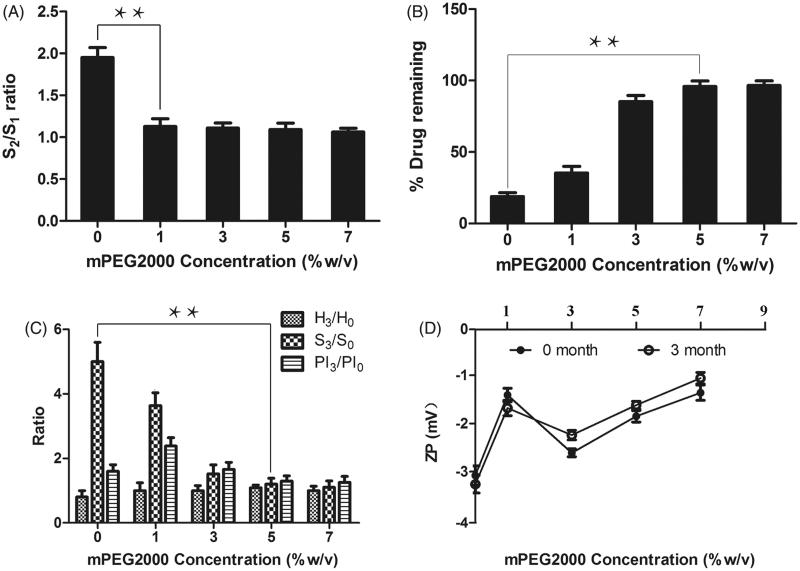
(A) Particle size (S_2_/S_1_) ratio of the CsA-loaded polymeric micelles composed of various concentration of mPEG2000. (B) Percent CsA remaining of polymeric micelles comprising various concentration of mPEG2000 after 3 months storage at 4 °C. (C) and (D) The measured parameters of the CsA-loaded polymeric micelles after 3 months storage at 4 °C compared with the initial time. H_3_/H_0_ is the ratio of height of lyophilized cake; S_3_/S_0_ is the ratio of particle size of polymeric micelles; PI_3_/PI_0_ is the ratio of polydispersity index of polymeric micelles; ZP is the Zate potential, the subscript numbers 0 and 3 denote 0 and 3 months after storage, respectively. Mean ± SD, *n* = 3. **Statistically significant difference between the compared formulations (*p* < .01). ***Statistically significant difference between the compared formulations (*p* < .001).

To assess the efficiency of mPEG2000 acting as a stabilizer, the redispersibility of lyophilized polymeric micelles was investigated. As shown in Figure 2(A), the absence of mPEG2000 in the CsA – loaded polymeric micelles caused the S_2_/S_1_ ratio nearby 2.0.

The addition of mPEG2000 (1%, 3%, 5% and 7% w/v) greatly decreased such ratio to lower than 1.2. The result indicated that 1% w/v mPEG2000 was sufficient to stabilize CsA-loaded polymeric micelles upon the lyophilization process.

### 3.4. Physical stability study of lyophilized CsA-loaded polymeric micelles

After storing at 4 °C for 3 months, the physical stability of lyophilized polymeric micelles was evaluated in terms of the changes in particle size, polydispersity index, zeta potential, and height of lyophilized cake. The results were compiled in Figure 2(C,D). The height of lyophilized cake, particle size and polydispersity index of polymeric micelles were expressed as the ratio after 3 months storage compared with an initial time.

The height ratio of most formulation after 3 months storage was unchanged. However, the formulation without a stabilizer could not be redispersed to obtain the same size and size distribution to the original ones. The stability of the CsA-loaded polymeric micelles directly depended on the concentration of mPEG2000. The formulations without mPEG2000 showed 5 times larger size than the initial size. The increasing mPEG2000 concentration led to smaller size after reconstitution. The addition of mPEG2000 at the concentration of 5% (w/v) resulted in the CsA-loaded polymeric micelles with unchanged particle size and size distribution as compared to those at the initial. The particle size of CsA micelles greatly increased after storage for 3 months probably due to the precipitation of drugs during storage. Nevertheless, the mPEG2000 concentration at 5% and 7% w/v could prevent the physical instability of CsA-loaded polymeric micelles. The zeta potential (ZP) values of the CsA-loaded polymeric micelles were not affected by storage at 4 °C for 3 months.

On the other hand, TEM micrographs (Figure 1(E)) indicated that lyophilized CsA micelles with 5% w/v mPEG2000 were still spherical in shape and monodispersed with no aggregations after 3 months storage at 4 °C. From these results, the concentration of mPEG2000 could affect the physical stability of CsA-loaded polymeric micelles and the minimum concentration of mPEG2000 was 5% w/v.

### 3.5. Chemical stability study of lyophilized CsA-loaded polymeric micelles

The formulations containing 1%, 3%, 5% and 7% w/v mPEG2000 were selected to evaluate their chemical stability in comparison with those without mPEG2000. The results were demonstrated in Figure 2(B). The increasing mPEG2000 concentration led to larger % CsA remaining after 3 months storage at 4 °C. When the mPEG2000 concentration at 5% and 7% w/v, the % CsA remaining of the CsA formulations was higher than 95% as compared to that at the initial time. The result indicated that the minimum concentration of mPEG2000 was 5% w/v. Combining the results of physical and chemical stability tests, it can be concluded that the micelles with 5% w/v mPEG2000 were suitable for further development.

### 3.6. Rheological studies

**Figure 3.**
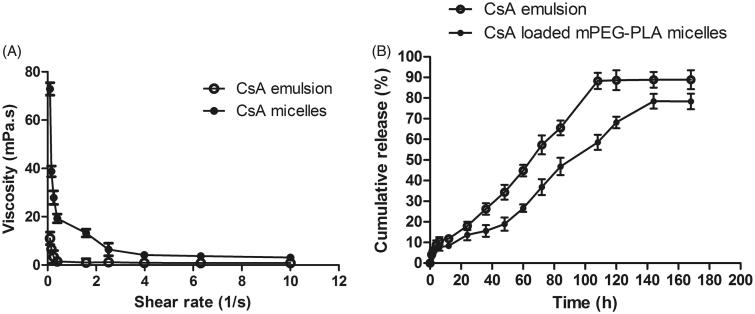
(A) Rheology profiles of different formulations; (B) in vitro release profile of CsA emulsion and CsA loaded mPEG-PLA micelles in 0.25% SLS solution. Mean ± SD, *n* = 3.

Figure 3(A) showed the rheology of different formulations. The viscosity of CsA micelles were much higher than CsA emulsion (*P* < 0.05), and they exhibited pseudoplastic rheology, as shown by a decrease in viscosity with increasing shear rate. The administration of ophthalmic preparations should have minimal effect due to the pseudoplastic character on the precorneal film. The ocular shear rate was very large, ranging from 0.03 1/s during interblinking periods, 4250–28500 1/s during blinking. The ophthalmic preparation with a viscosity that is high under low shear rate conditions and low under high shear rate conditions were often preferred (Geethalakshmi et al., 2012).

### 3.7. *In vitro* release kinetics study

To evaluate CsA release profiles from the emulsion and drug-loaded micelles, *in vitro* release study was carried out in 0.25% SLS solution. As depicted in Figure 3(B), CsA released from micelles were slower than those from emulsion. The CsA release process for micelles and emulsion were 7-day long with 78.36% and 88.87% of drug release, respectively. The release profile of CsA-loaded micelles was gentle and did not show a burst effect. The reason was that there was a strong interaction between the drugs and the core of the micelles (Huang et al., 2014). Therefore, it was difficult for the drug to be released from the core, which also resulted in a slow release rate. This effect prolonged action time and reduced the administration times.

In order to determine the mechanism of CsA release from emulsion and micelles, the release data was evaluated by model-dependent methods. The results were shown in Supplementary Table S3. There was a good linear relationship between the drug cumulative release and the time (R > 0.99), illustrating that the preparation had a sustained release effect.

CsA release from emulsion and micelles followed Weibull mechanism, which implied that the drug release was dependent on diffusion and swelling-erosion (Dou et al., 2014).

### 3.8. *In vitro* cytotoxicity assay

A promising nanoparticle system intended for ocular use must be capable of delivering sufficient levels of the active agent without comproming the viability of the host cells. Thus, it was interesting to study the effect of different CsA formulations on the viability of HCE-2 cell lines.

**Figure 4.**
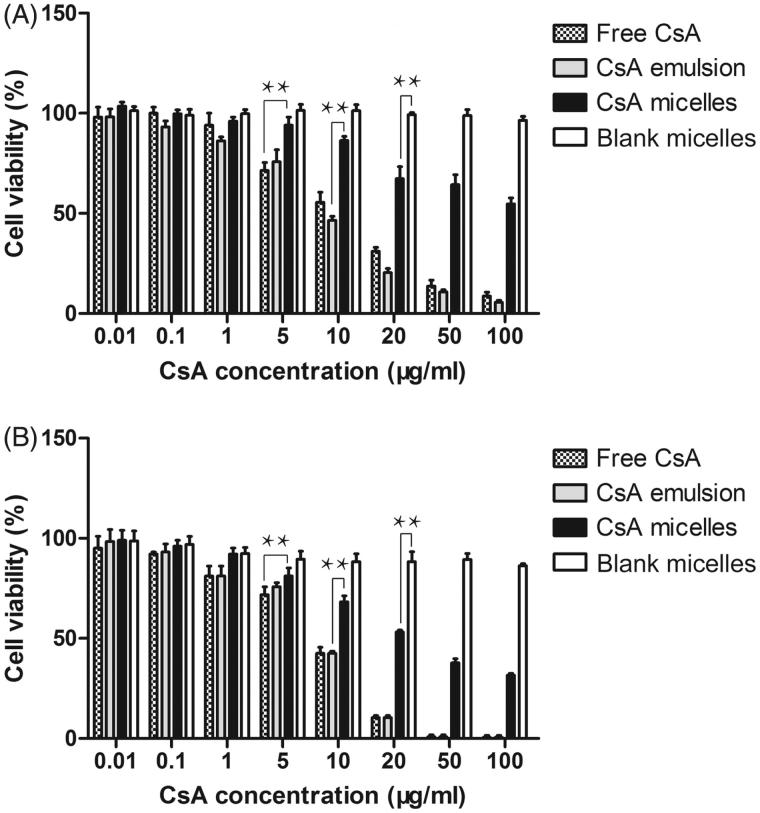
The cytotoxicity study on free CsA and CsA emulsion and CsA-loaded mPEG-PLA micelles. (A) and (B) represent the cytotoxicity of free CsA and CsA emulsion, CsA-loaded micelles and blank micelles against HCE-2 cells at 24 h and 48 h, respectively. *n* = 6, mean ± SD. *, **, and *** represents *p* < .05, *p* < .01 and *p* < .001, respectively.

The results of cell survival after treatment with different CsA formulations were presented in Figure 4. The survival rate of HCE-2 cells was decreased with increasing incubation time (*p* < .01). Cell viability was also decreased with increasing the CsA concentration applied (*p* < .01). CsA-loaded micelles appeared to be significantly less cytotoxic than free CsA (*p* < .05). The lower toxicity of CsA-loaded micelles was probably related to its vehicle, which delayed the release of CsA. At the end of the 24 h and 48 h experiments, mPEG-PLA blank micelles were nontoxic with a cell survival above 85% for CsA concentration ranging from 0.01 to 100 μg/ml. Although the survival percentage of the blank micelles was high, a low percentage cell death was thought to be related to the polymer or residue of organic solvent remaining from the preparation procedure.

### 3.9. Cellular uptake study

**Figure 5.**
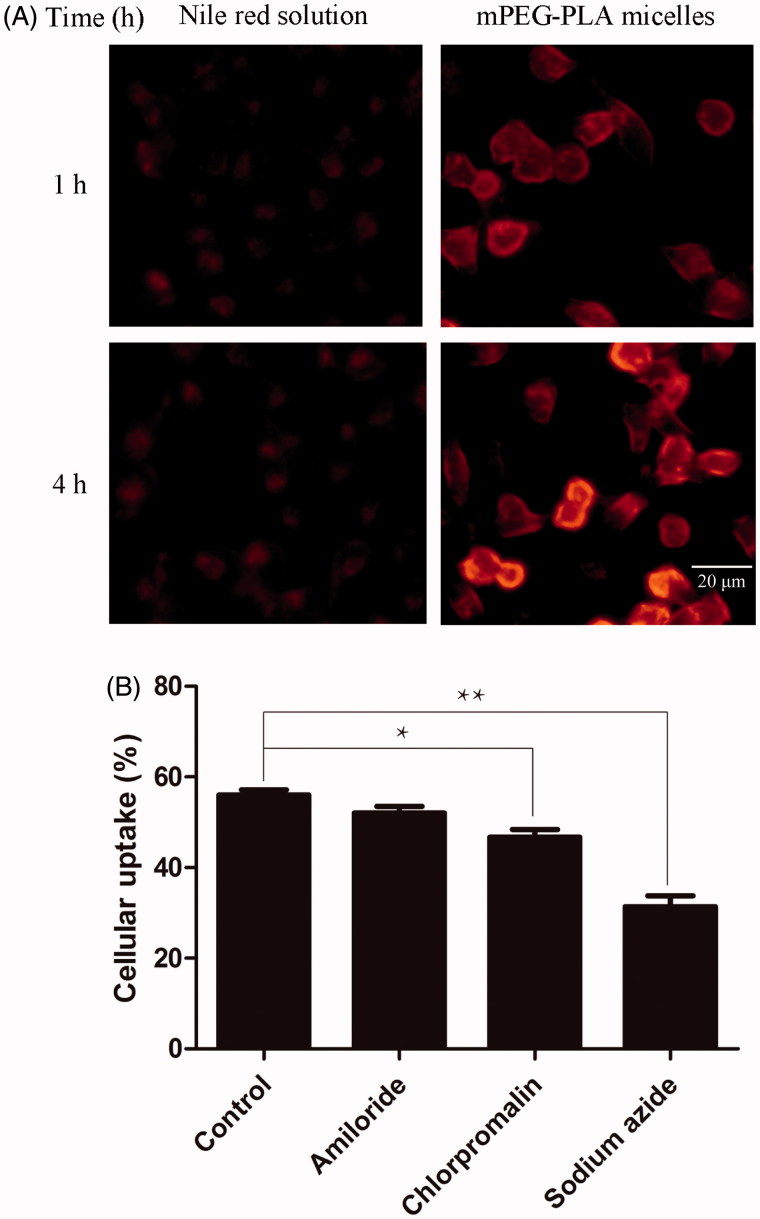
Cellular uptake of nile red by HCE-2 cells. (A) Photographs of representative series of cells exposed to 1 μl/ml nile red reference solution and mPEG-PLA micelles for 1 h and 4 h, respectively. (B) These histograms showed the cellular uptake efficiency (%) of different inhibitors on HCE-2 cells (*n* = 3, mean ± SD). * and ** indicated *p* < .05 and *p* < .01 versus control group.

The effect of mPEG-PLA micelles on the HCE-2 cellular uptake of nile red was studied by fluorescence inverted microscope. Nile red, which has low aqueous solubility, was used as a red fluorescent probe. As shown in Figure 5(A), an obvious time dependent increase in uptake amount was observed from 1 h to 4 h. Cellular uptake of nile red loaded in mPEG-PLA micelles showed higher fluorescence than those exposed in free nile red. It was conceivable that the positively charged particle surface facilitated ionic interaction with the negatively charged HCE-2 cells membrane, which cause the cellular uptake efficiency of nile red micelles was higher than that of nile red reference solution.

### 3.10. Cellular uptake mechanisms studies

Inhibitors of different mechanisms were chosen to investigate the cellular uptake of mPEG-PLA micelles (Figure 5(B)). Amiloride, the inhibitor of macropinocytosis mediated endocytosis (Koivusalo et al., 2010), did not significantly decrease the uptake of polymeric micelles. Chlorpromazine, the inhibitor of clathrin-mediated endocytosis (Perry & Wobus, 2010), significantly prevented the uptake of micelles (*p* < .05). The results showed that clathrin-mediated endocytosis participated in the micelles endocytosis. Sodium azide is the inhibitor of cell energy metabolism. After the pretreatment of sodium azide, the cellular uptake of micelles remarkably reduced compared to that of the control group (*p* < .01), which indicated the uptake of mPEG-PLA micelles was energy dependent.

### 3.11. Ocular distribution study

**Figure 6.**
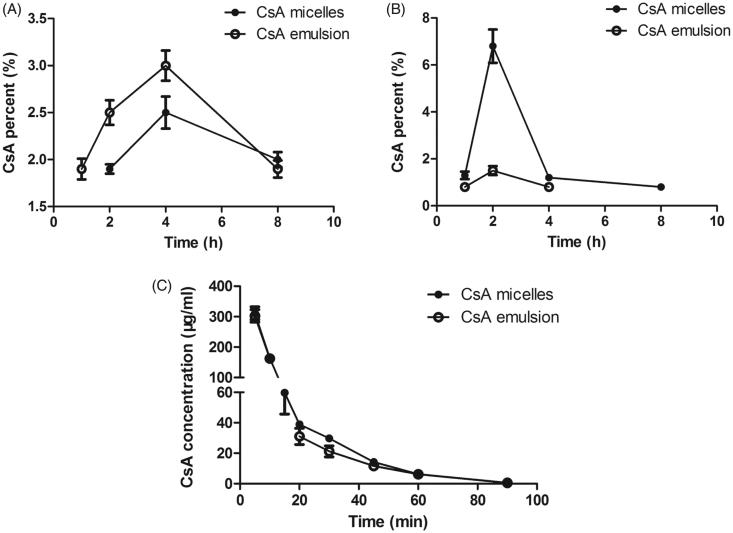
CsA percent-time profiles of CsA after instillation of CsA micelles and CsA emusion in the (A) aqueous humor and (B) corneal (*n* = 3, mean ± SD). (C) Concentration of CsA in rabbit tear fluid as a function of time after instillation of the CsA loaded mPEG-PLA micelles and 5% CsA emulsion (*n* = 6, mean ± SD).

The drug biodistribution in different rabbit ocular issues was shown in Figure 6(A,B). The corneal and aqueous humor retention after administration of CsA micelles were similar with CsA emulsion, and the concentration reached the maximum after administration 2 h and 4 h, respectively. It is obviously seen that the maximum concentration of CsA in the rabbit corneal after instillation of CsA loaded mPEG-PLA micelles was 4.5-fold higher than that of administration of CsA emulsion (*p* < .05) and there were still 0.8% CsA stranded at 8 h. The CsA micelles showed lower CsA levels in aqueous humor compared to the CsA emulsion (*p* < .05). The CsA micelles significantly prolonged the residence time on the ocular surface. As mentioned for the rheology results, high viscosity would prolong the residence time of formulations on the ocular surface. After topical administration, the drug first came into contact with the cornea, penetrated into the cornea, then penetrated through the cornea into the aqueous humor and other tissues. There were no blood vessels in the cornea, and the drug was absorbed by passive transport, so a higher concentration of drug within the cornea helps with drug absorption to other ocular tissues (González-Peñas et al., 1998). On the other hand, dry eye disease was a multifactorial disorder of the tears and ocular surface (Lemp, 2007). For corneal at each time point, the CsA concentration of CsA micelles was significantly higher than that of CsA emulsion, showing it could effectively contact with corneal, even prolonging the retention time of CsA on the corneal, producing effective dry eye treatment. In addition, side effects caused by systemic absorption were avoided.

### 3.12. The pharmacokinetic study of CsA ophthalmic in rabbit eye

The concentration of CsA in rabbit tear fluid as a function of time was monitored after application of 60 μl of CsA loaded mPEG-PLA micelle solution and 5% CsA emulsion into the rabbit eye. The concentration - time curve of CsA in the tear fluid after instillation of these formulations are shown in Figure 6(C). All data were carefully conducted by 3p87 software and Akaike’s information criterion (AIC) was compared. According to the AIC of different compartment models, the compartmental analysis indicated that the pharmacokinetic behavior of CsA in rabbit tear fluid was best fitted the two-compartment models due to the smallest AIC value. The main pharmacokinetic parameters of compartmental model were listed in Supplementary Table S4. The data fitness was evidenced by the high correlation (*R* > 0.999) between computer conducted and experimental CsA tear fluid concentrations.

The concentration-time curves of CsA micelles and emulsion were comparative coincidence. The pharmacokinetics of CsA micelles and emulsions in tears were divided into two phases, the first phase was quick elimination and the second phase was slow elimination. The rapid elimination should be related to the predominance of a strong and rapid precorneal drug loss linked to the interaction of CsA with the ocular structures and to trigger the specific protective mechanisms of the eye after the administration of the eyedrop to conjunctival sac (Rodriguez-Aller et al., 2012). The slow elimination was corresponded with a predominant loss of drug which was remained in the eye surface or corneal surface. In a global sense, the area under the curve (AUC) of CsA micelles and emulsions was statistically the same, but the distribution rate constant (α) and elimination rate constant (β) of two preparations showed statistically significant differences (*p* < .05). The α and β of CsA micelles were smaller than that of CsA emulsions, which indicated that the CsA elimination of micelles was slower than that of CsA emulsions from eyes and CsA micelles retained longer in the eye than CsA emulsion. This could be due to the elimination of CsA was enhanced by the irritation of the small molecule excipients (such as castor oil and Tween 80) used in emulsions. For 5% CsA emulsion, some adverse effects such as visual disturbance, ocular burning, redness or epiphora (Allergan, 2013) could be linked to the stimulation of the protective mechanisms of the eye, which could reinforce the elimination. Previous study has demostrated that the corneas and conjunctiva were covered by thin fluid layers named mucus films composed of negatively charged mucin (Sharma et al., 1999). Positively charged particles could possibly develop additional molecular attraction forces by electrostatic interactions with negatively charged mucin, because positive polymer and mucin are an attraction pair with different charges (He et al., 1998). The cationic characteristics of CsA micelles cause ionically interaction with the negatively charged mucus layer at the eye surface. Positive charged surface properties offer the prospects of prolonging the retention time of CsA micelles in the eye surface and consequently ensuring optimal contact between the formulation and the eye.

## 4. Conclusions

In the present study, the potential of mPEG-PLA micelles as a drug nano-carrier for ocular delivery was investigated. CsA, as a non-steroidal immunosuppressant drug being clinically used, was successfully encapsulated into mPEG-PLA micelles by a simple thin-film dispersion method. The lyophilized formulations containing 5% mPEG2000 were chemically and physically stable after storage at 4 °C for 3 months. Meanwhile, the *in vitro* release showed that CsA micelles had sustained and delayed release. The *in vitro* cytotoxicity study showed that mPEG-PLA blank micelles were nontoxic. The cellular uptake showed that mPEG-PLA micelles enter the cells via energy dependent by clathrin mediated endocytosis. Ocular distribution study demonstrated that CsA micelles was obtained better retention effect than 5% CsA emulsions. Furthermore, micelles more efficiently sustained the CsA concentrations in tear fluid and its elimination was slower as compared to 5% CsA emulsions. Therefore, these nano-micelles could represent a superior alternative to the currently applied oil-based CsA ophthalmic solution.

## Supplementary Material

IDRD_Shen_et_al_Supplemental_Content.doc
