# Supplementary material for: Improving the topical ocular pharmacokinetics of lyophilized cyclosporine A-loaded micelles: formulation, in vitro and in vivo studies
Source: Drug Deliv. 2018 Apr 10;25(1):888–99. doi: 10.1080/10717544.2018.1458923 (PMC6058700; doi:10.1080/10717544.2018.1458923)
Supplement: IDRD_Shen_et_al_Supplemental_Content.doc [file IDRD_A_1458923_SM8468.doc]

Supporting information

**Improving the topical ocular pharmacokinetics of lyophilized cyclosporine A - loaded micelles: formulation, in-vitro and in-vivo studies**

Yinglan Yu^a^, Daquan Chen^b^, Yanan Li^a^, Wenqian Yang^a^, Jiasheng Tu^a^*, Yan Shen^a^*

**Materials and methods**

***Characterization of CsA loaded micelles***

*Fourier transform infra-red (FTIR)*

FTIR spectra of mPEG-PLA co-polymer, CsA powder, physical mixture of CsA and mPEG-PLA as well as freeze dried CsA loaded mPEG-PLA micelles were obtained with Nicolet iS10 spectrophotometer (Thermo Fisher, USA), using the potassium bromide (KBr) disk technique (about 10 mg of sample for 100 mg dry KBr).

*X-Ray diffraction (XRD)*

For XRD analysis, an automatic X-ray diffractometer (D8 Advance X, Bruker, Germany) equipped with an X-ray generator was used. CuKα radiation of 20 mA in the range (2θ) of 5 to 60° was employed for determination.

*Differential scanning calorimetry (DSC)*

The thermal properties of the samples were characterized by a DSC (TA Instruments, USA). Samples of mPEG2000, mPEG-PLA co-polymer, CsA powder, physical mixture of CsA, mPEG-PLA and mPEG2000 as well as freeze dried CsA loaded mPEG-PLA micelles were separately sealed in alminum cells and heated from 25°C to 300°C at a heating rate of 10°C per minute in a nitrogen atmosphere. Alumina was used as the reference substance.

***The pharmacokinetic study of CsA micelles in rabbit eyes***

New Zealand white rabbits were feed to standard environmental conditions (25°C, RH 50%, and 12 h light/dark cycle). Food and water were supplied and libitum. Prior to study, fifteen rabbits were used to determine precorneal pharmcokinetics. Approximately 50 μl CsA micelles (0.5 mg/ml) was instilled on the right eye, and 50 μl CsA emulsion (0.5 mg/ml) was instilled on the left eye, respectively. At the predetermined time points, tears were collected by strips method . Briefly, at 5, 10, 15, 20, 30, 45, 60, 90 min after instillation, tear samples of both eyes were collected by Schirmer test strips. The amount of tear withdrawn was calculated by subtracting the weight of each strip after sampling from the weight before sampling. The eyelids were gently held to close during sampling in order to prevent loss of the eye drops. Subsequently, the Schirmer strip was placed into an Eppendorf tube, dried under N_2_ stream, and then 0.2 ml of mobile phase was added. The sample was vortexed for 30 s thoroughly to dissolve CsA into the mobile phase and centrifuged under 10000 rpm for 5 min. All the experiment were performed under the Protocol of Ethical Comittee Permission. The concentration of CsA in the supernatant was determined by HPLC and the amount of CsA remaining in per g of the tears was taken as the concentration of CsA in each time point.

The pharmacokinetic parameters associated to each animal were estimated by compartmental method using 3p87 Pharmacokinetic Program (Chinese Society of Mathematical Pharmacology 1987, China). The program showed a two compartment open model fitted to the drug concentration-time profiles with the lowest Akaike’s number.

**Results and discussion**

***Characterization of CsA micelles***

Table S1 Formulative variables of CsA loaded mPEG-PLA micelles (n = 3, mean ± SD)

| Block ratio (mPEG : PLA) | Mean diameter (nm) | Poly dispersity  Index | Zeta potential  (mV) | Encapsulation efficiency (%) |
| --- | --- | --- | --- | --- |
| 80 : 20 | 28.8 ± 2.4 | 0.44 ± 0.03 | 3.20 ± 0.98 | 62.26 ± 2.1 |
| 60 : 40 | 33.0 ± 1.6 | 0.52 ± 0.07 | 1.20 ± 0.86 | 76.38 ± 4.2 |
| 40 : 60 | 42.2 ± 3.2 | 0.66 ± 0.09 | 3.20 ± 0.12 | 98.03 ± 3.8 |

Table S2 Screening results of six excipients

| Excipients | Solution appearance | After standing for 12 h | Lyophilized product appearance |
| --- | --- | --- | --- |
| No excipients | Clear and transparent | Precipitation after 2 h | Intact and porous cake structure |
| Glucose | Precipitation | Precipitation | Slight collapse and cracks on the surface |
| Sucrose | Precipitation | Precipitation | Serious collapse on the around |
| L-glutamic acid | Precipitation | Precipitation | Slight collapse and cracks on the surface |
| Sorbitol | Clear and transparent | Precipitation after 6 h | Shrinking into a huddle |
| Mannitol | Clear and transparent | Precipitation after 4 h | Intact and porous cake structure |
| mPEG2000 | Clear and transparent | Clear and transparent | Intact and porous cake structure |

***Physicochemical characterizations***


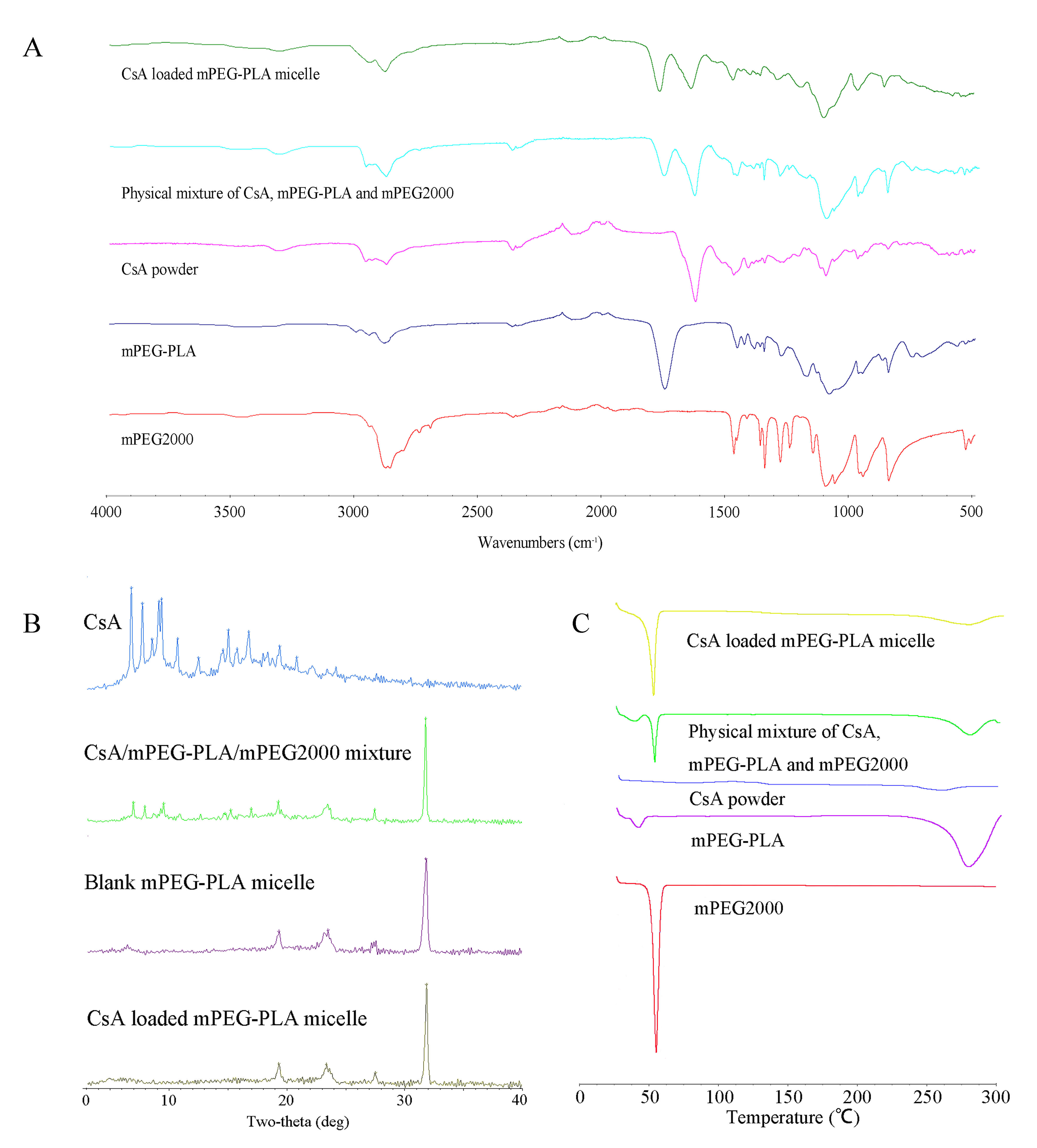


Fig. S1 (A) FTIR spectra of mPEG2000, mPEG-PLA, CsA powder, a physical mixture of CsA, mPEG-PLA and mPEG2000 and CsA loaded mPEG-PLA micelles. (B) XRD pattern of CsA powder, a physical mixture of CsA, mPEG-PLA and mPEG2000, blank mPEG-PLA micelles and CsA loaded mPEG-PLA micelles. (C) DSC profiles of mPEG2000, mPEG-PLA, CsA powder, a physical mixture of CsA, mPEG-PLA and mPEG2000 and CsA loaded mPEG-PLA micelles.

***In vitro release kinetics study***

Table S3 CsA emulsion and CsA loaded mPEG-PLA micelles release in vitro

| Model | CsA emusion |  | CsA micells |  |
| --- | --- | --- | --- | --- |
|  | Equation | R | Equation | R |
| Zero-order | Q=0.667t | 0.9729 | Q=0.516t | 0.9886 |
| First-order | Q=100[1-Exp(-0.012t)] | 0.9797 | Q=100[1-Exp(-0.008t)] | 0.9726 |
| Higuchi | Q=6.888t^0.5^ | 0.9707 | Q=5.205t^0.5^ | 0.9493 |
| Hixson-Crowell | Q=100[1-(1-0.004*t)^3^] | 0.9864 | Q=100[1-(1-0.002*t)^3^] | 0.9805 |
| Weibull | Q=100{1-Exp[-((t+26.320)^2.167^)/24643.286]} | 0.9943 | Q=100{1-Exp[-((t+32.874)^2.153^)/48670.244]} | 0.9943 |
| Peppas-Sahlin | Q=-1.765t^0.342^+3.347t^0.684^ | 0.9816 | Q=-0.977t^0.557^+0.229t^1.114^ | 0.9882 |

***The pharmacokinetic study of CsA ophthalmic in rabbit eye***

Table S4 Compartmental pharmacokinetics parameters of CsA in tear fluid after topical administration in the conscious rabbits (n = 6, mean ± SD)

| Pharmacokinetics parameters | Emulsion group | Micelles group |
| --- | --- | --- |
| α (1/min) | 0.22 ± 0.012^*^ | 0.19 ± 0.0089 |
| β (1/min) | 0.032 ± 0.0026^*^ | 0.025 ± 0.00088 |
| T_1/2_(min) | 21.66 ± 3.45 | 27.50 ± 2.88 |
| AUC (μg/g)*min | 5368.72 ± 145.62 | 5368.87 ± 138.84 |
| CL(s) μl/min/(μg/g) | 0.00011 ± 0.000022 | 0.00019 ± 0.00006 |

^*^P＜0.05 vs. Micelles group
